# Infant Feeding Practices of Emirati Women in the Rapidly Developing City of Abu Dhabi, United Arab Emirates

**DOI:** 10.3390/ijerph120910923

**Published:** 2015-09-02

**Authors:** Hazel Gardner, Katherine Green, Andrew Gardner

**Affiliations:** 1 School of Chemistry and Biochemistry, University of Western Australia, Crawley, Perth, WA 6009, Australia; 2 International Horizons College, 42nd Floor, U-Bora Towers, Al Abraj Street, Business Bay, Dubai P.O. Box 191881, United Arab Emirates; E-Mail: Katherine.Green@ihc-dubai.com; 3 School of Natural Sciences, Edith Cowan University, Joondalup, Perth, WA 6027, Australia; E-Mail: A.gardner@ecu.edu.au

**Keywords:** breastfeeding, complementary feeding, developing country, United Arab Emirates, infant feeding

## Abstract

Rapid economic and cultural transition in the United Arab Emirates (UAE) has been accompanied by new challenges to public health; most notably a rapid rise in chronic disease. Breastfeeding is known to improve health outcomes in adulthood, is associated with reduced risk of developing chronic disease, and is therefore an important public health issue for this rapidly increasing population. Factors associated with infant feeding practices were examined in a cohort of 125 Emirati women and their infants, with data collected at birth and 3, 6 and 15 months postpartum by questionnaires and interviews. Participants were recruited in the Corniche Hospital, the main maternity hospital in the city of Abu Dhabi. Factors affecting the duration of breastfeeding and the introduction of complementary foods were investigated using univariate and multivariate statistics. Recommended infant feeding practices, such as exclusive breastfeeding for the first six months of life and timely introduction of appropriate complementary foods, were poorly adhered to. Factors implicated in early cessation of breastfeeding included: time to first breastfeed, mother’s education level, employment status and early introduction of complementary foods.

## 1. Introduction

Breastmilk provides optimal nutrition for growth and development together with significant immunological protection [1,2] and exclusive breastfeeding is recommended for six months, and then alongside complementary feeding, well into the first year of life [3]. However exclusive breastfeeding until six months of age is a practice that is not widely followed in developed countries [2,4,5] and is even less common in developing countries [6,7].

The United Arab Emirates (UAE) is a rapidly modernising country with high levels of chronic disease; particularly obesity, heart disease and diabetes [8]. Infancy is a critical period in relation to nutrition, and breastfeeding has been shown to reduce the risk of chronic illness later in life [9].

Exclusive breastfeeding for the first six months of life is a global public health recommendation [10]. The UAE breastfeeding policy states that infants should be breast fed exclusively until six months of age [11]. In Islam breastfeeding is viewed as an expectation of parents, and women are urged to breastfeed their infants for two years [12]. As an Islamic country, the United Arab Emirates promotes breastfeeding throughout the health system and indeed in 2014 the country’s Federal National Council passed a draft clause in the child rights law to make breastfeeding mandatory for the first two years of an infant’s life [13].

Previous studies on exclusive breastfeeding in the UAE show conflicting results. The UAE Family Health Survey found that 34% of infants were exclusively breastfed to four months [14]. Another report documents 46% of mothers exclusively breastfeeding at 4–6 months [15]. However, Al Mazroui found that only 4% of infants in a cohort in the city of Al Ain were exclusively breastfed during the first month of life [16]. More recently a study on a multi-cultural population including Emiratis reported that exclusive breastfeeding rates at one and six months were 48% and 13% respectively [17].

The time of implementation of breastfeeding after birth is of crucial importance and has been shown to have an effect on infant health and the duration of breastfeeding [18,19]. UNICEF recommends that women should be encouraged to initiate breastfeeding within the first hour of birth [20]. The UAE Family Health Survey found that although 97% of women initiated breastfeeding in the hospital, only 23% did so in the first hour after birth and 29% did not begin breastfeeding until more than six hours after birth [14]. Similarly, Al Mazroui *et al.*, found that only 51% of mothers in Al Ain started breastfeeding on the first day after birth [16]. A more recent study suggests that the delay in time to first breastfeed has significantly decreased, with 82% of mothers feeding within the first hour postpartum. This is probably due to improved practices in hospitals in the UAE [21].

Education and employment have a negative impact on breastfeeding, particularly exclusive breastfeeding, in the UAE [14,21,22,23] and in other parts of the Arabian Gulf [24,25,26,27] which is congruent with developed countries such as USA and Australia, where maternal employment is associated with a reduction in duration of breastfeeding [28,29,30,31]. The level of education and employment amongst women in the UAE population is increasing, and this may lead to a further reduction in breastfeeding rates with potentially deleterious effects.

UNICEF recommendations are that infants should not be fed food or drinks other than breast milk in the neo-natal period, unless medically indicated [20]. However, introduction of complementary fluids and foods before six months is reportedly common in this population, particularly in working mothers and those with higher levels of education [14]. More recently Radwan found that 13.5% of infants had received complementary foods before 3 months and 83.5% before 6 months [21]. Early introduction of foods such as cow’s milk, eggs and honey may increase the risk of allergies and food poisoning [32,33].

In this population, where the birth rate is still relatively high, it has been found that birth order influences the length of time an infant is breastfed, with the first-born infant being fed for longer [17,21,23].

Given the dose dependent relationship for many of the benefits of breastfeeding for the infant few studies have investigated the prevalence and practices of breastfeeding into the second year of life in this population, although this appears to be relatively common compared to other countries [14,21]. According to the Emirates Family Health Survey 50% of infants were still receiving some breast milk at 12–15 months [14].

The aim of this study was to identify rates of breastfeeding at 3, 6 and 15 months postpartum in a cohort of Emirati women. In addition, factors associated with the duration of breastfeeding including early introduction of foods/drinks other than breastmilk and the reasons mothers stopped breastfeeding were examined.

## 2. Methods

This paper focuses on the data collected in relation to infant feeding practices, although the study encompassed a wide range of cultural, social, behavioural aspects of health in this cohort of women and infants.

125 Emirati women, together with their husbands/guardians, provided written, informed consent to participate in the study, which was approved by the Human Research Ethics Committee at Zayed University, Abu Dhabi, UAE. Questionnaires were designed following input from international consultants and Emirati female researchers, who ensured cross-cultural equivalence of the instruments [34]. All materials were created in English and then translated into Arabic using a cross-translation technique [35]. Under this technique an Emirati female research assistant translated the English document into Arabic, and then a different Emirati assistant (blind to the original document) retranslated the document back into English. Any differences identified were reviewed with Emirati and Western researchers and modified to minimise semantic differences.

A pilot study was conducted in which ten Emirati women, who had just given birth in the Corniche Hospital (government maternity hospital in Abu Dhabi), were recruited. As a result of this, further adaptations to the study were made in order to account for maternal literacy and the number of visitors in the mother’s hospital room.

All Emirati women who gave birth in the Corniche Hospital over the period of a month (October 2002) were invited to be part of the study. Around 10% of the eligible participants declined to take part in the study, primarily due to ill health or because they were refused permission from their male guardian. An Arabic-speaking female research assistant interviewed mothers during their postpartum stay in the hospital. Additionally their medical records were reviewed and then they were contacted via mail and/or telephone at three, six months and fifteen months postpartum.

The definition of exclusive breastfeeding followed the World Health Organisation definition, as recommended by Binns *et al.* 2009 (See Appendix 1) [36]. Data were analysed using IBM SPSS Statistics package Version 23 [37] (2015). Initially, univariate logistic regression analyses of two variables (food and drinks other than water introduced at three months; mother still breastfeeding at 15 months) were undertaken against the explanatory factors. Adjusted odds ratios and their 95% confidence limits were used to assess significant relationships.

Multivariate logistic regression analysis were undertaken to determine which factors were independently associated with the introduction of food and drink other than water at 3 months, and with the continuation of breastfeeding until 15 months. All relevant factors with sufficient data were included in the modeling. Backward stepwise elimination of factors was used, with removal based on the likelihood ratio test. In the final model, the only factors retained were those whose removal would result in a significant change in likelihood.

## 3. Results

The number of respondents decreased at each data collection. Initially there were 125 mothers and infants, but this reduced to 94 at three months, 58 at six months and 52 at 15 months. The participant characteristics are shown in Table 1.

**Table 1:** Characteristics of mothers & infants.

Participant Characteristics			
Maternal			
Age in years (mean, SD, range)	28.7	5.7	16–46
Age at marriage in years (mean, SD, range)	20.8	4.5	11–38
Parity (mean, SD, range)	3.4	2.1	1–9
Primiparous (n, %)	29	23	
Education level (n, %)			
None	6	5	
Primary	28	22	
Secondary	62	50	
Diploma/degree	29	23	
Working before birth (n, %)	36	29	
Consanguineous marriage (n, %)	60	48	
Polygamous marriage (n, %)	7	6	
Household			
Size of household (mean, SD, range)	7.9	4.4	3–24
Domestic helpers (mean, SD, range)	1.6	1.4	0–10
Infant			
Sex (n, %)			
Male	62	49.6	
Female	63	50.4	
Gestation in weeks (mean, SD, range)	39.1	2.4	25–44
Birthweight in kg (mean, SD, range)	3.2	0.6	0.7–4.4

Antenatal care is available free of charge to all Emirati women and all the mothers had at least one antenatal appointment at the maternity hospital. The majority of participants (93%, *n* = 116) initially planned to breastfeed their infants. Only one stated that she planned to feed infant formula and 8 of the women were undecided. One hundred and nineteen (95%) of the mothers breastfed their infants in the hospital, and four (3%) infants were fed infant formula only. Four of the eight infants in the neo-natal intensive care unit were also receiving breast milk and two infants who were extremely premature were receiving parenteral nutrition. Almost half of the women in the study (*n* = 59, 50%), breastfed their infant within the first hour after birth while 18% (*n* = 21) did not breastfeed their infant until more than 4 h after birth. The majority (*n* = 76, 61%) of mothers planned to breastfeed for more than 18 months. Only 9% of the mothers in this study intended to stop breastfeeding before their infant was six months of age. The prevalence of breastfeeding at each of the data collections is shown in Table 2.

**Table 2:** Prevalence of breastfeeding.

Breastfeeding ^a^	Birth		3 Months		6 Months		15 Months	
*n*	125		94		62		52	
	n	%	n	%	n	%	n	%
None	5	4	6	6	9	15	26	50
Complementary	25	20	46	49	53	85	26	50
Predominant	26	21	37	39	0	0	0	0
Exclusive	69	55	5	5	0	0	0	0

**^a^** Definitions following Binns *et al*. 2009 [36]. See Appendix 1.

Thirty (24%) of the participants stated that they did not plan to feed infant formula. The majority planned to combine breastfeeding with complementary feeding of infant formula. The reasons given for feeding formula included: to supplement breastfeeding (*n* = 42, 34%), for convenience when returning to work/studying (*n* = 24, 19%), and if the infant seemed hungry (*n* = 11, 9%).

It was common (*n* = 44, 35%) to feed substances other than breastmilk/infant formula during the first few hours and days postpartum. These were usually water or ritualistic in nature such as honey, herbal infusions or dates (see Table 3).

At three months, 42 of the participants were feeding only breast milk (45%, *n* = 94), 46 (49%) a mixture of breast milk and formula, while the remaining 6 (6%) were feeding only infant formula. However, further investigation showed that, of all the participants reporting they were solely breastfeeding, only five (5%) had not given the infant any other liquids or foods. The reasons given for feeding both formula and breast milk as a supplement to breastfeeding included; feeding the child in public places, for convenience after return of the mother to work/school, and when the infant was hungry or sick. At 3 months postpartum 49 (52%) of the women planned to breastfeed for eighteen to twenty-four months and only five (5%) planned to stop before their infant was 6 months of age. At three months postpartum 14 (15%) of the participants were expressing breast milk.

**Table 3:** Foods & drinks infants consumed by six months.

Food	Time Postpartum					
	1 week (*n* = 125)		3 months (*n* = 94)		6 months (*n* = 58)	
	n	%	n	%	n	%
Water	24	19	70	74	47	81
Tea	9	7	64	68	35	60
Cow/camel milk	-	-	24	26	21	36
Sugar water	12	10	24	26	20	34
Fruit juice	-	-	14	15	34	59
Honey	3	2	31	33	27	47
Dates	1	1	25	27	29	50
Infant food	-	-	18	21	31	53
Biscuits (rusks)	-	-	17	19	43	74
Fruit/vegetables	-	-	14	15	53	91
Yoghurt	1	1	13	14	38	66
Cereal	-	-	9	12	27	47
Rice	-	-	8	9	38	66
Meat	-	-	-	-	10	17
Eggs	-	-	-	-	18	31
Traditional dishes	-	-	-	-	15	26

Table 4 lists the variables considered which potentially may have an influence on whether a mother is supplementing the infant’s diet with foods and drinks other than water at three months. The univariate odds ratios indicate the likelihood that the mother is giving supplementary foods or drinks at three months postpartum. No associations were found between supplementing the diet at three months and socio-demographic factors including mother’s age, father’s age, mother’s age at marriage, consanguineous marriage, family size, and domestic help. Significant associations were found with whether the mother was currently working and with whether a pre-lacteal feed had been given. Currently working and the administration of pre-lacteal feeds increased the likelihood of having given supplementary food at three months.

**Table 4:** Factors influencing the likelihood of a mother giving supplementary food at 3 months postpartum.

Variables	Supplemental Food Given at 3 Months				OR	Lower 95% CI	Upper 95% CI
	YES		NO				
	n	%	n	%			
Mothers age							
<29	35	67	17	33	1.00		
≥29	27	64	15	36	0.87	0.37	2.01
Father’s age							
<31	31	67	15	33	1.00		
≥31	31	67	15	33	1.00	0.42	2.39
Mother’s age at marriage							
<21	26	59	18	41	1.00		
≥21	36	72	14	28	1.78	0.75	4.21
Consanguineous marriage							
yes	34	72	13	28	1.00		
no	28	60	19	40	0.56	0.24	1.34
Number of live births							
<4	39	70	17	30	1.00		
≥4	23	61	15	39	0.67	0.28	1.59
Birth mode							
Caesarean	47	65	25	35	1.00		
Vaginal	15	68	7	32	1.14	0.41	3.16
Infants gender							
male	33	72	13	28	1.00		
female	29	60	19	40	0.60	0.24	1.46
Infant weight							
<2.5 kg	3	75	1	25	1.00		
≥2.5 kg	59	66	31	34	0.63	0.06	6.36
Education level							
none or Primary	15	54	13	46	1.00		
Secondary	29	67	14	33	3.12	0.90	10.76
Tertiary	18	78	5	22	1.74	0.54	5.65
Mother currently working							
yes	22	88	3	12	1.00		
no	40	56	29	44	5.32 *	1.45 *	19.46 *
Time to first breastfeed							
<1 h	27	61	17	39	1.00		
1 to 3 h	21	64	12	36	2.94	0.73	11.76
>3 h	14	81	3	19	2.67	0.64	11.19
Feeding Plan							
Breastfeeding only	55	64	31	36	1.00		
Breastfeeding and formula	1	100	0	0	3.38	0.39	29.39
Not sure	6	86	1	14			
Infant received pre-lacteal feed							
yes	33	85	6	15	1.00		
no	29	53	26	48	0.20 *	0.07 *	0.56 *
Rooming in							
yes	56	64	31	36	1.00		
no	6	86	1	14	3.32	0.38	28.86
Hospital staff showed mother how to breastfeed							
yes	43	68	20	32	1.00		
no	19	63	11	37	0.80	0.32	2.00
Hospital staff recommended feeding on demand							
yes	50	66	26	34	1.00		
no	12	67	6	33	1.04	0.35	3.09
Family size							
Small, 6 or fewer	30	73	11	27	1.00		
Large, 7 or more	32	60	21	40	0.56	0.23	1.35
Number of wives							
1	58	66	30	34	1.00		
2	4	67	2	33	1.03	0.18	5.97
Primiparous							
yes	15	68	7	32	1.00		
no	47	65	25	35	0.88	0.32	2.43
Number of domestic helpers							
none	4	50	4	50	1.00		
1	35	66	18	34	2.86	0.56	14.60
>1	20	74	7	26	1.58	0.57	4.35

Number (%) and univariate odds ratios (95% confidence intervals) for supplemental food given at 3 months (*n* = 94). The Odds Ratio indicates the likelihood of a mother giving supplementary food at 3 months postpartum. Odds Ratios significantly different from 1 are marked with an *.

These two variables were also found to be independently associated with supplemental feeding at three months using a multivariate logistic regression model produced after backward stepping elimination of non-significant factors (Table 5). Currently working increased the likelihood of having supplemented the infant’s diet at three months by a factor of seven times, and pre-lacteal feeding increased the likelihood of having supplemented the infant’s diet at three months by a factor of about five times.

The third data collection point took place when infants were around six months of age, and by this time, the number of respondents had decreased to 58. An additional four women were identified as breastfeeding at six months as they reported breastfeeding at 15 months bringing the known total to 62. At six months all the infants had been given some form of solid foods, especially the three most popular introductory foods of fruits and vegetables (the most common were potatoes, carrots, squash, apples, and bananas), rice and cereal. Two of mothers had breastfed infants other than their own and one infant had been breastfed by another woman.

**Table 5:** Multivariate binomial modelling: factors independently associated with supplementary feeding given at three months and still breastfeeding at 15 months after adjustment for potential confounding variables.

Multivariate Binomial Regression Model	Variable	Yes/No	n	Adjusted OR	Lower 95% CI	Upper 95% CI
Supplementary feeding at 3 months **^a^**	Currently working	yes	65	1.00		
		no	23	7.46	1.55	35.88
	Pre-lacteal feed given	yes	37	1.00		
		no	51	0.182	0.06	0.56
Mother still breastfeeding at 15 months **^b^**	Supplementary feeding at three months	yes	32	1.00		
		no	16	4.58	1.19	17.67

**^a^** Non-significant variables were: mother’s age, father’s age, mother’s age at marriage, consanguinity, number of live births, birth mode, infants gender, hospital staff taught breastfeeding methods, hospital staff recommended breastfeeding on demand, education level, time to first breastfeed, rooming in; **^b^** Non-significant variables were: mother’s age, father’s age, mother’s age at marriage, consanguinity, number of live births, birth mode, infants gender, hospital staff taught breastfeeding methods, hospital staff recommended breastfeeding on demand, pre-lacteal feed given, education level, time to first breastfeed, mother currently working, rooming in.

The fourth data collection was conducted when infants were aged 14–15 months and the number of respondents at this stage had reduced to 52. Twenty-six (50%) of these participants were still breast-feeding and seventeen of them were feeding only breast milk along with complementary foods. The 17 mothers feeding only breast milk all planned to continue until two years. Two of the mothers who were employed were still giving breast milk at 14–15 months. Factors associated with breastfeeding at 15 months are analysed in more detail in Table 6.

Table 6 lists variables that may potentially have an influence on whether a mother is still breastfeeding 15 months postpartum. The univariate odds ratios indicate the likelihood of a mother still breastfeeding at 15 months postpartum. No associations were found with socio-demographic factors including mother’s age, father’s age, mother’s age at marriage, and consanguineous marriage. Variables associated with breastfeeding at 15 months were mother’s education level, mother currently working, time to first breastfeed, and whether the infant received supplemental food and drinks other than water at three months. Mothers were less likely to be breastfeeding at 15 months if they have an education level greater than primary, if they are working, if the infant received its first breastfed more than an hour after birth, and if the infant received supplemental food or drink at three months.

The final model resulting from multivariate binomial regression with backward stepping to remove non-significant variables, resulted in a single variable remaining: whether the infant received supplemental food and drinks other than water at three months (Table 5). This indicates considerable covariance between the factors. The odds of a mother still breastfeeding at 15 months were over 4 times greater if the infant did not receive supplemental feeding at three months.

**Table 6:** Factors influencing the likelihood of mothers still breastfeeding at 15 months.

Variables	Still Breastfeeding at 15 Months				OR	Lower 95% CI	Upper 95% CI
	YES		NO				
	n	%	n	%			
Mothers age							
<29	13	45	16	55	1.00		
≥29	13	57	10	43	1.60	0.53	4.82
Father’s age							
<31	11	46	13	54	1.00		
≥31	15	56	12	44	1.48	0.49	4.46
Mother’s age at marriage							
<21	14	58	10	42	1.00		
≥21	12	43	16	57	0.54	0.18	1.62
Consanguineous marriage							
yes	11	46	13	54	1.00		
no	15	54	13	46	1.36	0.46	4.07
Number of live births							
<4	13	42	18	58	1.00		
≥4	13	62	8	38	2.25	0.72	6.99
Birth mode							
Caesarean	5	36	9	64	1.00		
Vaginal	21	55	17	45	0.45	0.13	1.60
Infants gender							
male	14	50	14	50	1.00		
female	12	50	12	50	1.00	0.34	2.98
Infant weight							
<2.5 kg	1	25	3	75	1.00		
≥2.5 kg	25	52	23	48	3.26	0.32	33.61
Education level							
none or Primary	11	69	5	31	1.00		
Secondary	12	52	11	48	0.14 *	0.03 *	0.72 *
Tertiary	3	23	10	77	0.28	0.06	1.27
Mother currently working							
yes	2	18	9	82	1.00		
no	24	59	17	41	0.16 *	0.30 *	0.82 *
Time to first breastfeed							
<1 h	13	65	7	35	1.00		
1 to 3 h	11	52	10	48	0.12 *	0.02 *	0.71 *
>3 h	2	18	9	82	0.20	0.04	1.17
Infant received prelacteal feed							
yes	8	38	13	62	1.00		
no	18	58	13	42	2.25 *	0.72 *	6.99 *
Infant receiving supplemental foods at 3 months							
yes	12	38	20	63	1.00		
no	11	69	5	31	3.67	1.02	13.14
Rooming in							
yes	24	52	22	48	1.00		
no	1	25	3	75	0.31	0.03	3.16
Hospital staff showed mother how to breastfeed							
yes	16	48	17	52	1.00		
no	9	53	8	47	1.20	0.37	3.86
Hospital staff recommended feeding on demand							
yes	22	52	20	48	1.00		
no	3	38	5	63	0.55	0.12	2.58
Family size							
Small, 6 or fewer	10	45	12	55	1.00		
Large, 7 or more	16	53	14	47	1.37	0.46	4.14
Primiparous							
yes	7	27	19	73	1.00		
no	9	35	17	65	1.44	0.44	4.70

Number (%) and univariate odds ratios (95% confidence intervals) for any breastfeeding at 15 months (*n* = 52). The Odds Ratio indicates the likelihood of a mother still breastfeeding at 15 months postpartum. Odds Ratios significantly different from 1 are marked with an *.

The reasons mothers gave for cessation of breastfeeding over all four data collections are reported in Table 7.

**Table 7:** Reasons for cessation of breastfeeding.

Reason for Cessation of Breastfeeding	n	%
Insufficient/perceived insufficient milk	19	32
Infant feeding difficulties	7	12
Pregnancy	7	12
Poor infant weight gain	6	10
Mother decided it was appropriate time to stop	6	10
Infant illness	6	10
Maternal work/education	4	7
Nipple pain	2	3
Mother taking contraceptive pill	1	2
Maternal illness	1	2

## 4. Discussion

The UAE Ministry of Health recommends that infants should be exclusively breastfed until six months of age [11]. In addition, the Qur’an (2:233) advocates breastfeeding for the first two years of life. However, the findings from this study showed that very few infants were exclusively breastfed until three months of age. Ninety-five per cent of the Emirati women participating in this study initiated breastfeeding after birth while only four infants were being fed infant formula. These figures are similar to those reported by the UAE Family Health Survey where 97% of mothers in the Emirate of Abu Dhabi initiated breastfeeding at birth, and also to a more recent study which reported 98% of women initiated breastfeeding [14,21]. It appears, therefore, that initiation rates for breastfeeding are being maintained. However, many of the infants were not breastfed until several hours after the birth. This is concerning as it is important to establish breastfeeding as soon as possible after birth [18,38] to achieve full milk production. Half of the infants in this study were breastfed in the first hour after birth. In 1995 only 23% of infants in the UAE were breastfed within an hour of birth, but this has dramatically improved, with more recent studies reporting 75%–80% [14,17,21]. Herbal remedies and certain foods such as honey and dates are widely used by this population (Table 3) as they are thought to have health benefits. Consumption of these foods and drinks begins at an early age and is associated with early introduction of complementary foods (Table 6). Cultural significance may make it difficult to eradicate this practice, but it should be minimised to encourage longer periods of full breastfeeding.

It is common for mothers in the UAE to combine breast and formula feeding, and in this study only 24% (*n* = 30) of the mothers stated that they would not feed infant formula at birth. As reflected in Table 2, the majority planned to combine breast and formula feeding, since many believe that breastfeeding is insufficient, and supplementation with infant formula provides additional nutrition. Other reasons included the need to feed the infant when out in public, as many of the participants felt breastfeeding in public was culturally prohibitive and suitable facilities were often non-existent. Early supplementation has been widely reported in previous studies, and appears to have increased since the UAE Family Health Survey when 36% of infants were given infant formula along with breastmilk before 3 months postpartum [14]. At three months postpartum 55% (*n* = 52) had received some infant formula (Table 2). In other UAE studies the supplementation rate ranged between 14% and 53% [21,23]. Consistent with other studies [39,40], early introduction of complementary foods and drinks was found to be negatively associated with breastfeeding duration (Table 4 and Table 6). This is reflected in the reasons for ceasing breastfeeding, the most common being the mothers’ perception that they were producing insufficient milk to meet the infant’s requirements. Other reasons given for cessation of breastfeeding included: infant feeding issues, poor infant weight gain and pregnancy. These findings support other studies on Emirati populations [14,21]. The reasons for breastfeeding cessation were very similar to those expressed by mothers in other countries [24,41,42,43,44]. Supplementation may negatively impact milk supply due to infrequent suckling [45], which may lead to further supplementation with infant formula. This suggests that, in addition to increased education and awareness about the benefits of exclusive breastfeeding, information on the harmful effects of the early introduction of complementary foods may also be useful for new mothers.

Education and employment had a negative effect on breastfeeding in this study with infants of working mothers less likely to be breastfed and more likely to be introduced to complementary foods by 3 months (Table 5 and Table 6). Emirati women are becoming increasingly well educated, with over 70% of young women graduating at college or university level [46]. Higher levels of educational attainment are accompanied by expectations that they will join the workforce. The impact of maternal education and especially employment on infant feeding is not an issue unique to the Emirates, and has been widely reported both in the Middle East and other regions [23,26,30,31,40]. This may replicate a trend seen previously in developed countries where breastfeeding was seen as old fashioned. It may also be a result of the extensive advertising and promotion of follow-on milks in the UAE. This suggests that there will be an increasing need for support for working mothers if breastfeeding rates are to be maintained or improved.

Substantial numbers of the Emirati women participating in this study did continue to feed infants some breast milk throughout the duration of the study. At fifteen months postpartum, 50% of the infants remaining in the study were still receiving breast milk (Table 2). Therefore, although many of the mothers supplemented breastfeeding with infant formula or other foods, a significant number of these infants were receiving breastmilk for a longer duration than in many other cultures.

By six months postpartum the infants in this study had all been introduced to complementary foods. Very early introduction of eggs and dairy products are of concern, the level of food allergies among children in the UAE is estimated to be 5%–7%, with allergies related to consumption of cows milk being more prevalent [47]. In this study 22 (38%) of the infants had been fed milk other than breast milk or formula (*i.e*., cow/camel) by the age of six months, 38 infants (66%) had consumed yogurt and 18 (31%) eggs. Although there is conflicting evidence [48,49] in regard to the timing of the introduction potentially allergenic protein foods, exposure to small amounts may be beneficial in preventing allergies in the long term.

Given the high rates of obesity, diabetes and heart disease in the UAE population [8] and the potentially protective effect of breastfeeding [50] it is not surprising that the UAE leadership is attempting to encourage breastfeeding through a variety of measures including legislation. As the country continues to modernize it is important that Emirati women are provided with the support and education they need to breastfeed their infants, a practice that is embedded in their traditional and religious beliefs. Breastmilk provides complete nutrition and significant immunological protection [1,2,3] and this need to be emphasized to dispel the myth that infants require supplementation.

## 5. Limitations of the Study

The UAE was a challenging arena in which to undertake cross-cultural research as neither the hospital nor the Emirati families were accustomed to accommodating researchers. There were also difficulties posed by working in two languages, although the cross-translation technique [35] used overcame the more obvious problems.

The exploratory descriptive nature of this study depicts a unique set of circumstances documented at a single point in time, in the period prior to rapid development in Abu Dhabi. This, combined with the relatively small number of subjects, serves to limit any claims that this case study might make about representativeness. Caution is also required in extending generalisations from these findings to the circumstances of women outside the UAE. Even within the UAE, caution is urged, since the social, economic and cultural conditions found in other emirates are different from those in Abu Dhabi, as for example, the significantly larger population of Shi’ite Muslims, as compared with Sunni Muslims, living in the Emirate of Ras al-Khaimah.

The subject attrition in the follow up data collections was partially attributable to difficulties in reconnecting with the subjects: many women tended to move between residences, and to change their phone numbers. In addition, since it was essential that the Emirati women’s husbands agreed to their participating in the study, patriarchal influences may have inhibited women from participating, who might have otherwise identified important issues worthy of researching.

Finally, Emirati women who gave birth to sick infants or who were ill themselves were unlikely to have volunteered to take part in this study at such a stressful time. Hence, these subjects with more serious health issues were not represented in the study, and should be considered an excellent focus for future research.

## 6. Conclusions

Emirati mothers experience many of the same barriers and influences to breastfeeding as women in other countries. The challenge for leaders in this rapidly developing society is to develop policies and support mechanisms to ensure that women can maintain breastfeeding whilst continuing to play an important role in the development of their country.

## Appendix 1

**Table A1:** Criteria for inclusion in WHO infant feeding categories [36].

Category of Infant-Feeding	Requires That the Infant Receive	Allows the Infant to Receive	Does not Allow the Infant to Receive
Exclusive breastfeeding	Breast milk (including milk expressed or from wet nurse)	Drops, syrups (vitamins, minerals, medicine)	Anything else
Predominant breastfeeding	Breast milk (including milk expressed or from wet nurse) as the predominant source of nourishment	Liquids (water, and water-based drinks, fruit juice, ORS *****), ritual fluids and drops or syrups (vitamins, minerals, medicines)	Anything else (in particular, non-human milk, food-based fluids)
Complementary breastfeeding	Breast milk and solid or semi-solid foods	Any food or liquid including non-human milk	
Breastfeeding	Breast milk		
Bottle-feeding	Any liquid or semi- solid food from a bottle with nipple/teat	Also allows breast milk by bottle	

***** ORS, Oral Rehydration Solution.
